# Gut microbiota combined with fecal metabolomics reveals the effects of FuFang Runzaoling on the microbial and metabolic profiles in NOD mouse model of Sjögren’s syndrome

**DOI:** 10.1186/s12906-023-04017-5

**Published:** 2023-06-13

**Authors:** Changming Chen, Ping Zeng, Xueming Yao, Zhaowei Huang, Yi Ling, Ying Huang, Lei Hou, Hufan Li, Dan Zhu, Wukai Ma

**Affiliations:** Department of Rheumatology and Immunology, the Second Affiliated Hospital of Guizhou University of Traditional Chinese Medicine, Guiyang, China

**Keywords:** Sjögren’s syndrome, NOD mice, FuFang Runzaoling, Gut microbiota, Metabolomics

## Abstract

**Objective:**

Sjögren’s syndrome (SS) is an inflammatory autoimmune disease characterized by high levels of chronic lymphocyte infiltration. Differences and dysfunction in the gut microbiota and metabolites may be closely related to the pathogenesis of SS. The purpose of this study was to reveal the relationship between the gut microbiota and metabolome in NOD mice as a model of SS and the role of FuFang Runzaoling (FRZ), which is a clinically effective in treating SS.

**Methods:**

NOD mice were gavaged with FRZ for 10 weeks. The ingested volume of drinking water, submandibular gland index, pathologic changes of the submandibular glands, and serum cytokines interleukin (IL)-6, IL-10, IL-17 A, and tumor necrosis factor-alpha (TNF-α) were determined. The roles of FRZ on gut microbiota and fecal metabolites were explored by 16 S rRNA gene sequencing and liquid chromatography-mass spectrometry (LC-MC), respectively. The correlation between them was determined by Pearson correlation analysis.

**Results:**

Compared with the model group, the drinking water volume of NOD mice treated with FRZ increased and the submandibular gland index decreased. FRZ effectively ameliorated lymphocyte infiltration in the small submandibular glands in mice. Serum levels of IL-6, TNF-α, and IL-17 A decreased, and IL-10 increased. The *Firmicutes*/*Bacteroidetes* ratio in the FRZ treatment group was higher. FRZ significantly downregulated the relative abundance of the family *Bacteroidaceae* and genus *Bacteroides*, and significantly upregulated the relative abundance of genus *Lachnospiraceae_UCG-001*. Orthogonal projections to latent structures discriminant analysis (OPLS-DA) revealed the significant change in fecal metabolites after FRZ treatment. Based on criteria of OPLS-DA variable influence on projection > 1, *P* < 0.05, and fragmentation score > 50, a total of 109 metabolites in the FRZ-H group were differentially regulated (47 downregulated and 62 upregulated) compared to their expressions in the model group. Kyoto Encyclopedia of Genes and Genomes pathway analysis revealed enriched metabolic of sphingolipid metabolism, retrograde endocannabinoid signaling, GABAergic synapse, necroptosis, arginine biosynthesis, and metabolism of histidine, alanine, aspartate, and glutamate. Correlation analysis between gut microbiota and fecal metabolites suggested that the enriched bacteria were related to many key metabolites.

**Conclusions:**

Taken together, we found FRZ could reduce the inflammatory responses in NOD mice by regulating the gut microbiota, fecal metabolites, and their correlation to emerge a therapeutic effect on mice with SS. This will lay the foundation for the further studies and applications of FRZ, and the use of gut microbiotas as drug targets to treat SS.

**Graphical Abstract:**

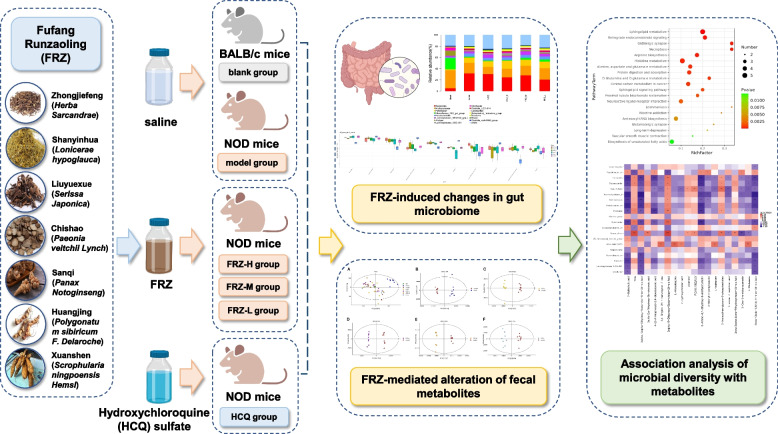

**Supplementary Information:**

The online version contains supplementary material available at 10.1186/s12906-023-04017-5.

## Introduction

Sjögren’s syndrome (SS) is a chronic and systemic autoimmune disease characterized by active B cell production of antibodies and lymphocyte infiltration of the exocrine glands [1]. The salivary and lacrimal glands of patients with SS are attacked, reducing the production of saliva and tears, which leads to dryness of the mouth and eyes, and swollen mumps. Further development of the disease also causes symptoms of fatigue and joint pain [2]. The pathogenesis of SS is unclear, although recent studies have closely related the pathogenesis of SS with the gut microbiota.

The gut microbiota and its metabolites can affect the host immune system in various ways. Gut microbiota disorders can lead to the destruction of the intestinal mucosal barrier and immune disorders, increase the release of inflammatory factors, and induce chronic inflammation, furthermore causing the occurrence and development of SS [3, 4]. Therefore, dysbiosis of the gut microbiota may play a role in the pathogenesis of SS. Metabolomics has been widely used for the diagnosis and monitoring of disease progression. The data have provided important insights into disease pathogenesis [5]. Non-targeted fecal metabolomics studies have clarified the understanding of the metabolites associated with gut microbiota alteration in the development of complex disease [6]. Knowledge of the gut microbiota combined with fecal metabolomics could help reveal the effects of drugs on microbial and metabolic profiles in organisms.

FuFang Runzaoling (FRZ) is composed of Zhongjiefeng (*Herba Sarcandrae*), Shanyinhua (*Lonicerae hypoglauca*), Liuyuexue (*Serissa Japonica*), Chishao (*Paeonia veitchii Lynch*), Sanqi (*Panax Notoginseng*), Huangjing (*Polygonatum sibiricum F. Delaroche*), and Xuanshen (*Scrophularia ningpoensis Hemsl*). This herbal preparation has therapeutically valuable actions that include relieve heat and dryness, detoxification, alleviating blood circulation, and promoting fluid production. Specifically, we have used FRZ to alleviate or decrease the clinical symptoms and inflammatory indicators of SS in patients [7, 8]. In addition, FRZ can strengthen the secretory function of glands and reduce the levels of inflammatory cytokines [9]. One hundred and nineteen potential targets of FRZ in regulating SS have been identified using network pharmacology techniques [10]. Preliminary experimental evidence has also confirmed that FRZ can regulate the expression of aquaporin 1 (AQP1) and AQP5 in the salivary gland tissue of NOD mice and improve water intake and submandibular gland index [11]. NOD mice are commonly used as an experimental animal model of SS. The pathological changes that occur in the submandibular glands of NOD mice are similar to those in SS. In addition, FRZ regulates the serum balance of Th17/T regulatory (Treg) cells and downregulates Th1/Th2 cells in NOD mice [12, 13]. However, an overall understanding of the interaction among FRZ and the gut microbiome is poor, and metabolomics data are scant.

In the present study, we applied 16 S rRNA high-throughput sequencing and fecal metabolomics to explore the changes in the gut microbiota and their metabolites in NOD mice, and investigate the regulatory role of FRZ. Our aim was to investigate the mechanism of FRZ in treating SS from the perspective of gut microbiota.

## Materials and methods

### Preparation of experimental drugs

FRZ comprised of 30 g Zhongjiefeng, 10 g Shanyinhua, 10 g Liuyuexue, 10 g Chishao, 5 g Sanqi, 15 g Huangjing, and 10 g Xuanshen was provided by the Chinese Pharmacy of the Second Affiliated Hospital of Guizhou University of Traditional Chinese Medicine. These herbal medicinal constituents were soaked in water, decocted, and filtered until the concentration of the liquid was 1.8 g/ml (The mouse equivalent dose was calculated based on the body surface area from FDA draft guidelines [14].). This concentration was considered as the high dose of FRZ. The medium dose (0.9 g/ml) and low dose (0.45 g/ml) of FRZ were obtained through equal dilution from the high dose FRZ. To control the quality of FRZ, HPLC analysis was performed on FRZ aqueous solution, and the HPLC analyses were performed on Shimadzu LC 20AT (Shimadzu, Japan). Hydroxychloroquine (HCQ) sulfate tablets were produced by Shanghai Shangyao Zhongxi Pharmaceutical Co., Ltd. (Shanghai, China; Batch No: H19990263; 1 g per tablet). HCQ is recommended for the diagnosis and treatment of SS based on the British Rheumatology Society and Chinese Rheumatology Society guidelines [15]. Appropriately, HCQ was the positive control in this study.

### Animals and experimental design

The NOD mouse model of spontaneous SS [16] was used for the experiments. Forty specific pathogen-free (SPF) female NOD mice and eight SPF female BALB/c mice (all 8-weeks-of-age with a body weight of 18–23 g) were obtained from Beijing Vitalstar Biotechnology Co., Ltd. (Beijing, China). The mice were housed in a temperature-controlled room at 25 ± 2 °C. They were provided common food and water during the experiments. This study was reviewed and approved by the Animal Ethics Committee of the Second Affiliated Hospital of Guizhou University of Traditional Chinese Medicine (Ky2019012 and Ky20190107). After acclimatization for 2 weeks, the eight BALB/c mice comprised the untreated (blank) group, and the forty NOD mice were randomly divided into five groups (*n* = 8 per group): model group, HCQ group, high dose FRZ group (FRZ-H; daily oral gavage with 18 g crude FRZ/kg), medium dose FRZ group (FRZ-M; daily oral gavage with 9 g crude FRZ/kg), and low dose FRZ group (FRZ-L daily oral gavage with 4.5 g crude FRZ/kg). Mice in the HCQ treatment group were orally gavaged daily with 40 mg/kg HCQ. Mice in the blank control and model groups were orally gavaged daily with saline (0.2 ml/10 g body weight). After 10 weeks of treatment, mice in all groups were fasted. The animals were sacrificed by cervical dislocation after collecting the serum, and tissues of submandibular glands were isolated and used for histopathological observation and subsequent experiments.

### Determination of general indicators

The volume of drinking water ingested by mice in each group was determined at the same time every week. The submandibular gland index was determined as submandibular gland weight (mg) / body weight (g). Pathological changes in the submandibular glands were observed using hematoxylin and eosin (H&E) staining. Images of the stained tissues were visualized by light microscopy using a model FSX100 microscope (Olympus Corporation, Japan).

### Determination of cytokine IL-6, IL-10, IL-17, and tumor necrosis factor-alpha (TNF-α) cytokines in serum

Blood samples were centrifuged at 1000 × g at 4 °C for 15 min, and the supernatants were collected for ELISA. The levels of serum cytokines IL-6, IL-10, IL-17 A and TNF-α were determined using an ELISA kit (Lianke, Hangzhou, China), according to the manufacturer’s protocol and instructions.

### Fecal DNA extraction and 16 S rRNA gene sequencing

Genomic DNA (gDNA) was extracted from mouse fecal samples using the QIAamp 96 PowerFecal QIAcube HT Kit (QIAGEN, Beijing, China) according to the manufacturer’s protocol. The quantity and quality of gDNA were determined using a NanoDrop2000 spectrophotometer (Thermo Fisher Scientific, USA) and agarose gel electrophoresis. The gDNA was diluted to 1 ng/µl and utilized as the template for PCR of the 16S rRNA gene. The variable V3-V4 region of 16S rRNA was amplified by PCR using universal primers (341F:5’-TACGGRAGGCAGCAG-3’ and 798R:5’-AGGGTATCTAATCCT-3’). The conditions of PCR were 94℃ for 5 min, followed by 26 cycles at 94℃ for 30 s, 56℃ for 30 s, and 72℃ for 20s, and a final extension at 72℃ for 5 min. PCR amplicons were extracted from agarose gels, according to the manufacturer’s instructions. After purification using AMPure XP beads (Beckman Coulter, USA) and quantification using Qubit (Thermo Fisher Scientific, USA) and the second round of PCR was performed. The Qubit dsDNA assay kit (Invitrogen, USA) was used to quantify the final purified PCR amplicon. Finally, according to standard protocols. Equal amounts of purified PCR amplicons were pooled and paired-end sequenced (2 × 250) using the MiSeq platform (Illumina, USA).

### Fecal metabolomics

Fecal metabolomics was detected using a Nexera ultra-performance liquid chromatography (UPLC) (Shimadzu, Japan) tandem QE high-resolution mass spectrometer (Thermo, USA). Fecal material (30 mg) in a 1.5 ml tube was mixed with two small steel beads, 0.3 ml methanol-water (4:1, v/v), and 10 µl internal standard (2-chloro-l -phenylalanine in methanol, 0.3 mg/ml) by 10 s of vortexing. After pre-cooling at − 40℃ for 2 min, the samples were ground in a grinder operating at 60 Hz for 2 min. The mixture was ultrasonicated in an ice-water bath for 10 min, kept at − 40 ℃ for 30 min, and then centrifuged at 14,300 × g and 4℃ for 10 min. Aliquots (0.2 ml) of supernatant were dried in a frozen concentration centrifugal dryer. Methanol (0.3 ml) and 1.2 ml of water were added, followed by vortexing for 30 s, ultrasonication for 3 min, and storage at − 20℃ for 2 h. Samples were centrifuged at 14,300 × g at 4℃ for 15 min. Aliquots (0.2 ml) of the clear supernatant from each tube was transferred to LC vials after filtered through 0.22 μm microfilters. Quality control (QC) samples were obtained by mixing aliquots of all samples. All samples were analyzed in both negative and positive ion modes. The ACQUITY UPLC HSS T3 column (100 mm × 2.1 mm, 1.8 μm; Waters, USA) and the guard column (Waters, USA) were utilized at 45 °C column temperature. The injection volume in both the negative and positive ion mode was 2 µl. The binary gradient elution system consisted of solvent A with 0.1% formic acid (Merck, Germany) in water and solvent B with 0.1% formic acid in acetonitrile (Merck, Germany) in the gradient elution mode (0–2 min, 95% B; 2–4 min, 95 − 70% A; 4–8 min, 70 − 50% A; 8–10 min, 50 − 20% A; 10–15 min, 0% A; 15–16 min, 95% A). The flow rate of the mobile phase was 0.35 ml/min, the mass range was from m/z 100 to 1,200, resolution of full mass spectrometry (MS) scans was 70,000, and resolution of higher-energy collisional dissociation tandem MS (HCD MS/MS) scans was 17,500. The collision energies were 10 eV, 20 eV, and 40 eV. MS conditions were spray voltage, 3,000 V (−) and 3,500 V (+); sheath gas flow rate, 40 arbitrary units; auxiliary gas flow rate, 10 arbitrary units; and capillary temperature, 320 °C.

### Integration analysis between modified gut microbiota and metabolites

All differential genera (one-way analysis of variance [ANOVA], *P* < 0.05) and significantly changed metabolites (variable influence on projection [VIP] > 1 and *P* < 0.05) were selected. Pearson correlation coefficients between bacterial genera and metabolites were calculated (^*^ *P* < 0.05, ^**^ *P* < 0.01).

### Statistical analysis

Statistical significance was analyzed using the Prism software (version 6.0; GraphPad Software Inc., USA). ANOVA and non-parametric tests were used to detect statistical significance among three or more groups; *P* < 0.05 was considered significant. Data are presented as mean ± SD. For metabolite data, LC-MS raw data were analyzed using analysis base File Converter software. Unsupervised principal component analysis (PCA), supervised partial least squares-discriminant analysis (PLS-DA), and orthogonal PLS-DA (OPLS-DA) were analyzed using SIMCA-P software. The differential metabolites were evaluated by VIP values of the OPLS-DA model > 1.0, and two-tailed Student’s t-test *P* < 0.05. The Kyoto Encyclopedia of Genes and Genomes (KEGG; http://www.kegg.jp/) website was used to analyze differential metabolite pathways [17, 18]. The association of fecal metabolite intensities with 16 S rRNA levels was tested using Spearman rank correlation.

## Results

### FRZ can alleviate SS pathogenesis in NOD mice

**Fig. 1:**
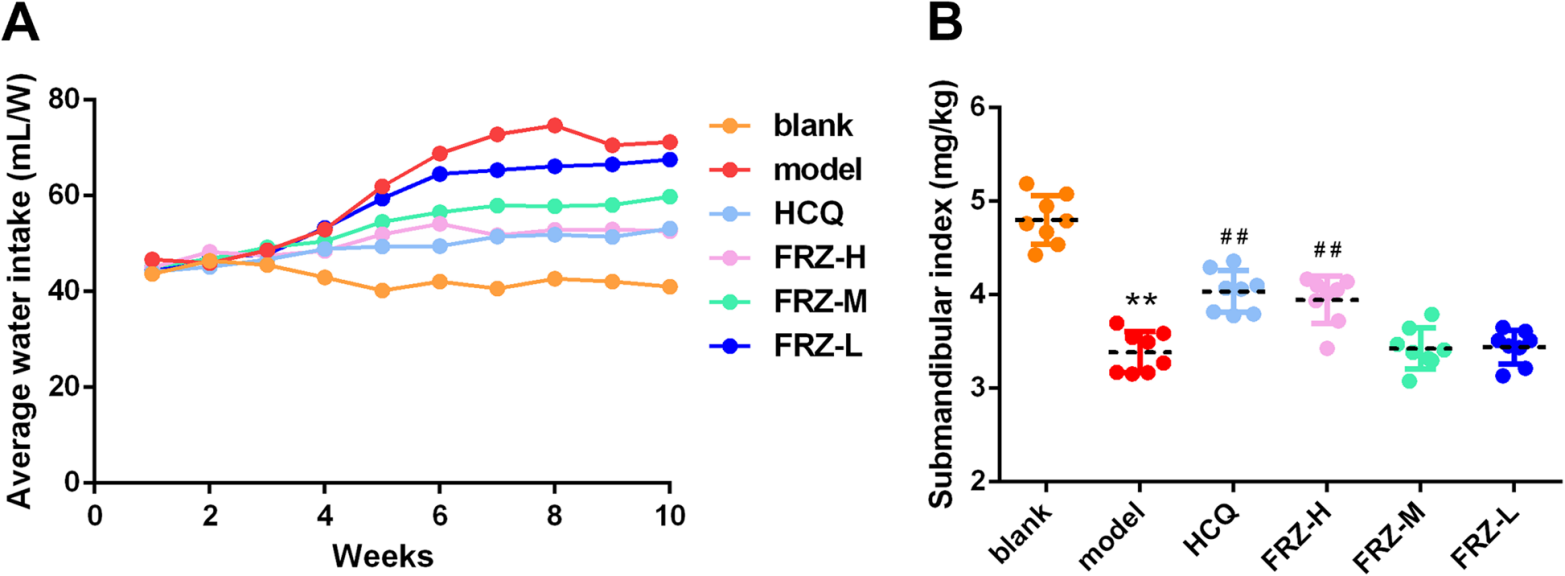
General indicators of FRZ for NOD mice. **A** The average water intake of mice in blank, model, HCQ, FRZ-H, FRZ-M and FRZ-L treatment groups from 1–10-weeks-of-age. **B** Submandibular gland index of mice in blank, model, HCQ, FRZ-H, FRZ-M and FRZ-L treatment groups

The average water intake of the mice and submandibular gland index in each group are shown in Fig. 1A and B. The average water intake of mice in the model group increased, while the submandibular gland index decreased compared with the blank group. The volume of drinking water of mice in the FRZ treatment and HCQ groups was decreased, while the submandibular gland index was increased compared to the model group.

**Fig. 2:**
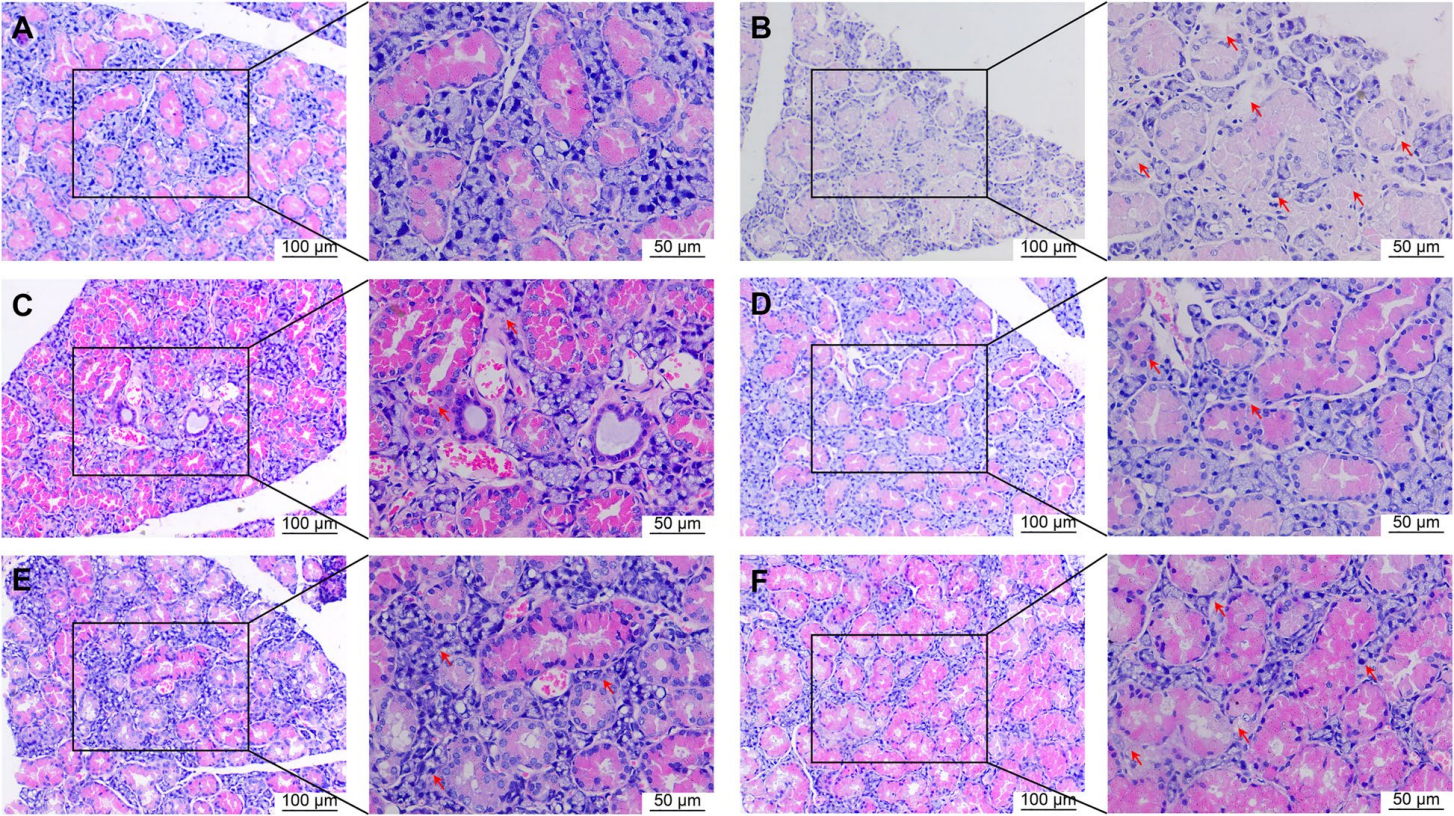
H&E staining of submandibular glands of mice in the blank (**A**), model (**B**), HCQ (**C**), FRZ-H (**D**), FRZ-M (**E**) and FRZ-L (**F**) treatment groups. Red arrows indicate the tissue infiltration and abnormal ordering of the acini and ducts. Scale bar = 100 μm (magnification, ×200) in the left panel and 50 μm (magnification, ×400) in the right panel. The cytoplasm of submandibular gland tissue cells is stained red, and nuclei are stained blue

H&E staining of submandibular glands is shown in Fig. 2. In the blank group, submandibular acini were uniform, orderly arranged, and interspersed with acinar ducts. The lobular structure of submandibular glands disappeared, acini and ducts were disordered, and local water-like degeneration of epithelial cells appeared in the model group. In addition, epithelial cells dissolved, small blood vessels around the acini were hyperplastic, dilated, and congested, and a small number of plasma cells and lymphocytes were observed between cells. In the FRZ-H treatment and HCQ groups, the lobular structure of submandibular glands was evident, acini and ducts were slightly disordered, and part of the acinar epithelium showed cell hyperplasia and water-like degeneration. In the FRZ-M and FRZ-L groups, the lobular structure of the submandibular glands disappeared, acini and ducts showed disordered arrangement, and water-like changes appeared in some epithelial cells.

### FRZ decreases serum proinflammatory cytokine levels of IL-6, TNF-α, IL-17 A, and increases the anti-inflammatory levels of IL-10

**Fig. 3:**
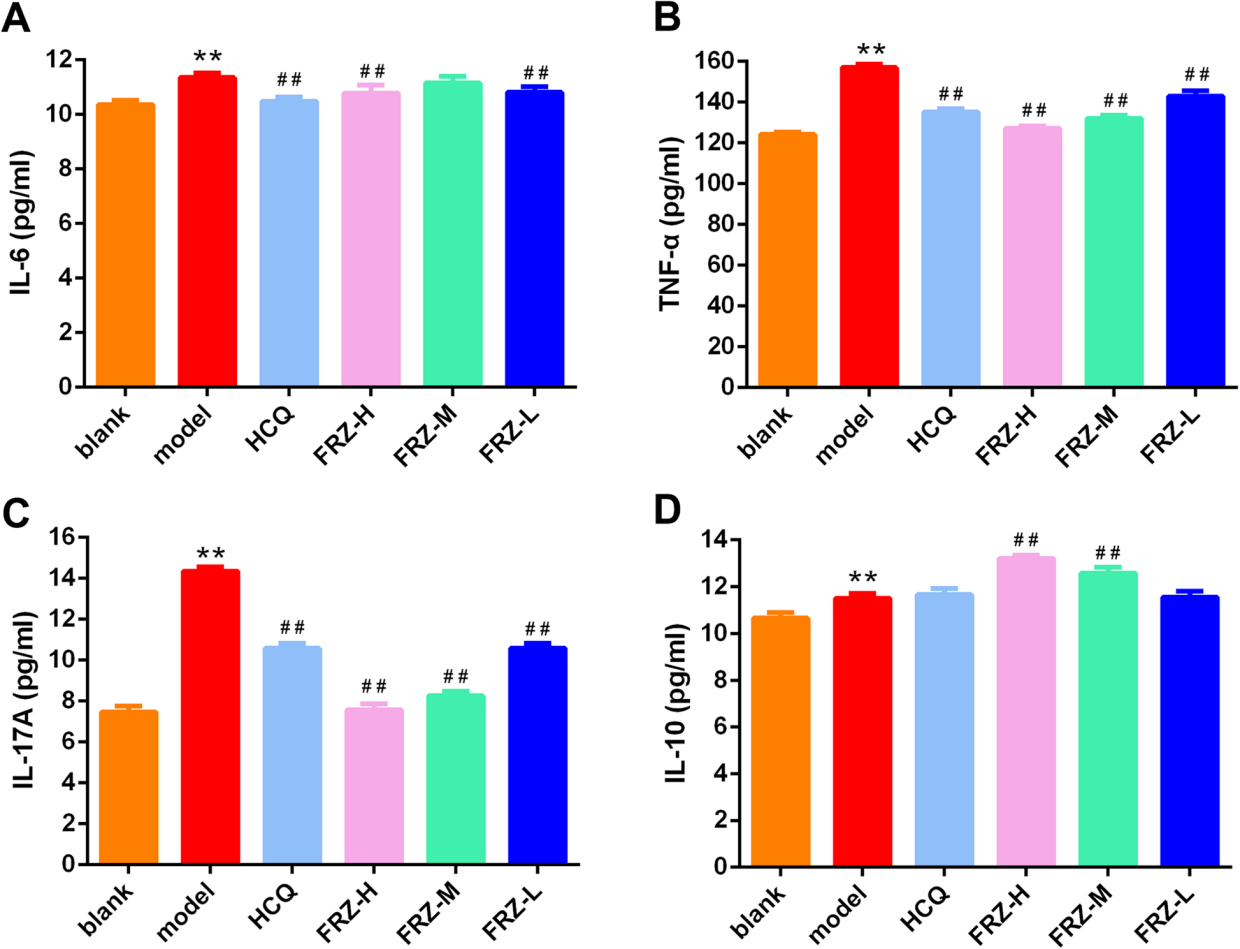
Serum proinflammatory cytokine levels of IL-6 (**A**), TNF-α (**B**), IL-17 A (**C**), and anti-inflammatory levels of IL-10 (**D**) in NOD mice. All data are expressed as mean ± SD. Significance levels in the model group versus blank group are ^*^ *P* < 0.05, ^**^ *P* < 0.01, and in the drug treatment group versus model group are ^#^ *P* < 0.05, ^##^ *P* < 0.01 (*n* = 8)

The proinflammatory cytokine levels of IL-6, TNF-α, IL-17 A, and IL-10 increased significantly in the model group compared to those in the blank group (all *P* < 0.01). The secretion of IL-6, TNF-α, and IL-17 A was significantly lower in the HCQ group than that in the blank group (all *P* < 0.01). The secretion levels of IL-6, TNF-α, and IL-17 A decreased significantly in the FRZ-H treatment group compared with those in the model group (all *P* < 0.01), while IL-10 was significantly increased (*P* < 0.01) (Fig. 3).

### FRZ‑induced changes in gut microbiome

**Fig. 4:**
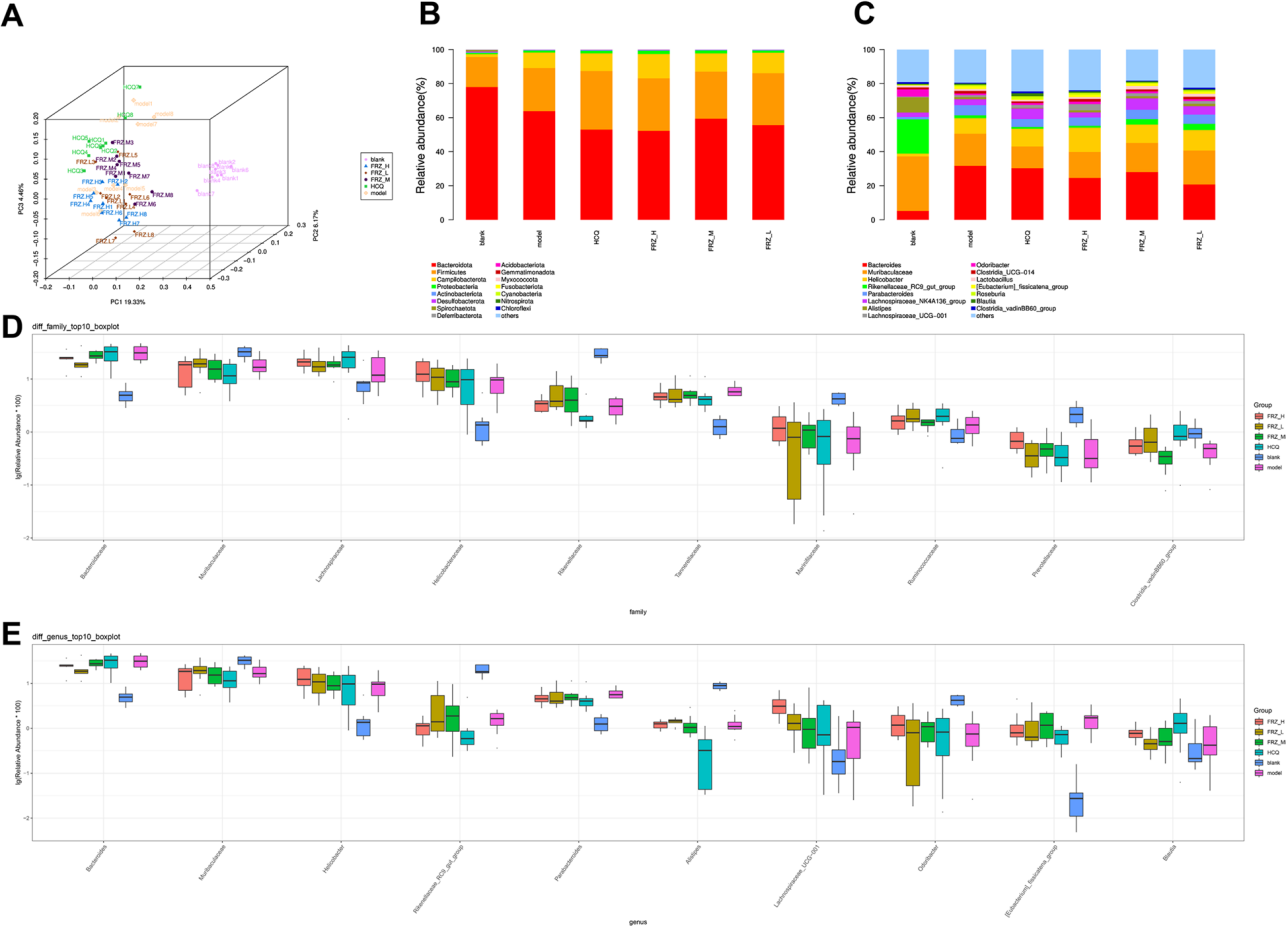
Assessments of differences in gut microbiota. PCoA of the gut microbiota in mice after FRZ treatment for 10 weeks (**A**) and the taxonomic distributions of bacteria (Top 15) at the phylum (**B**) and genus **C**. Scatter plots of significantly altered bacterial taxa, including family (**D**) and genus **E**. ^*^ *P* < 0.05 and ^**^ *P* < 0.01 according to ANOVA (*n* = 8)

Forty-eight samples were used to evaluate the microbial community profiles. Sequencing yielded a total of 47,835–74,592 clean reads, of which 43,058–67,368 were valid reads. The α-diversity indices are shown in Table 1, including Chao1, Observed species, Shannon, and Simpson indices. These indicators showed statistically significant differences between the blank and model groups, indicating that the diversity was significantly higher than that of the blank group. Compared with the model group, none of the above indices were disturbed by treatment with FRZ. Furthermore, we assessed whether there were differences in the microbiota composition between the six groups. Principal coordinate analysis (PCoA) on the unweighted UniFrac distances was performed to analyze differences in the gut microbial community structure. As shown in Fig. 4A, there was clear separation between the blank group and the other five groups, and the model group could be separated from the HCQ and FRZ-H groups. However, the model group could not be clearly distinguished from the FRZ-M and FRZ-L groups. These findings indicate that both HCQ and FRZ-H influenced the gut microbial composition and ameliorated the gut microbiome of NOD mice to a certain extent.

**Table 1:** α-diversity indices of mice gut microbiota in different groups

Group (*n* = 8)	Chao1	Observed species	Shannon	Simpson	Coverage
blank group	3042 ± 81.56	2266 ± 57.29	7.54 ± 0.12	0.96 ± 0.004	0.98
model group	2583 ± 126.10^**^	1742 ± 95.71^**^	6.24 ± 0.30^**^	0.92 ± 0.02^*^	0.98
HCQ group	2548 ± 90.82	1728 ± 74.28	6.24 ± 0.24	0.91 ± 0.02	0.98
FRZ-H group	2769 ± 58.90	1920 ± 53.52	6.44 ± 0.18	0.93 ± 0.01	0.98
FRZ-M group	2609 ± 72.68	1784 ± 66.12	6.40 ± 0.17	0.94 ± 0.01	0.98
FRZ-L group	2841 ± 79.73	1953 ± 81.58	6.79 ± 0.26	0.95 ± 0.01	0.98

Results are expressed as *x* ± *s*; compared with the blank group

^*^ *P* < 0.05

^**^ *P* < 0.01

### FRZ regulates gut microbial structure and function in NOD mice

The top 15 relatively abundant bacteria at the phylum and genus levels, according to taxonomic assignments, are shown in Fig. 4B C. At the phylum level, 90% of the sequences were within the top four phyla *Bacteroidetes*, *Firmicutes*, *Epsilonbacteraeota*, and *Proteobacteria*. Compared with the model group (63.81%), the proportion of *Bacteroidetes* in the FRZ treatment groups decreased, accounting for 52.25% (FRZ-H), 59.42% (FRZ-M), and 55.61% (FRZ-L), respectively. *Firmicutes* increased in the FRZ treatment groups, accounting for 30.84% (FRZ-H), 27.56% (FRZ-M), and 30.51% (FRZ-L) compared with the model group (25.33%). *Campilobacterota* increased in the FRZ treatment groups, accounting for 14.27% (FRZ-H), 10.81% (FRZ-M), and 12.04% (FRZ-L), compared with the model group (9.13%). Compared to the model group (1.17%), the proportion of *Proteobacteria* in the FRZ treatment groups gradually increased, accounting for 1.97% (FRZ-H), 1.68% (FRZ-M), and 1.27% (FRZ-L), respectively. Metastat analysis revealed significant differences in the relative abundance at the family and genus levels among the groups (Table 2; Fig. 4D and E). Compared with the blank group, the relative abundances of *Bacteroidaceae* (family), *Helicobacteraceae* (family), *Tannerellaceae* (family), *Bacteroides* (genus), *Helicobacter* (genus), *Parabacteroides* (genus), and *[Eubacterium]_fissicatena_group* (genus) in the model group were significantly increased, whereas the relative abundances of *Muribaculaceae* (family), *Prevotellaceae* (family), *Muribaculaceae* (genus), *Rikenellaceae_RC9_gut_group* (genus), *Alistipes* (genus), and *Odoribacter* (genus) in the model group decreased significantly. Compared with the model group, FRZ significantly downregulated the relative abundance of *Bacteroidaceae* (family) and *Bacteroides* (genus) and significantly upregulated the relative abundance of *Lachnospiraceae_UCG-001* (genus).

**Table 2:** Relative abundance (%) of blank, model, HCQ, FRZ-H, FRZ-M, and FRZ-L groups (*n* = 8 per group)

Taxonomy	blank (%)	model (%)		HCQ (%)	FRZ-H (%)	FRZ-M (%)	FRZ-L (%)
*Bacteroidaceae* (family)	5.13 ± 0.68	32.29 ± 3.81^**^		30.68 ± 4.33	24.70 ± 2.41	27.94 ± 1.94	21.52 ± 3.29^#^
*Muribaculaceae* (family)	32.30 ± 2.81	18.87 ± 2.83^**^		13.07 ± 2.49	15.90 ± 3.12	17.24 ± 3.02	20.87 ± 3.61
*Lachnospiraceae* (family)	9.03 ± 2.08	16.59 ± 3.82		24.66 ± 4.84	21.44 ± 2.60	18.81 ± 1.94	19.83 ± 3.35
*Helicobacteraceae* (family)	1.68 ± 0.57		9.09 ± 1.90^**^	10.18 ± 2.85	14.04 ± 2.67	10.73 ± 1.83	11.99 ± 2.63
*Rikenellaceae* (family)	29.72 ± 2.41		3.12 ± 0.44	2.11 ± 0.45	3.38 ± 0.35	4.96 ± 1.26	5.78 ± 1.42
*Tannerellaceae* (family)	1.32 ± 0.18		6.13 ± 0.54^**^	4.72 ± 0.97	4.94 ± 0.65	5.59 ± 0.90	5.46 ± 1.04
*Marinifilaceae* (family)	4.32 ± 0.36		0.92 ± 0.27	1.06 ± 0.34	1.43 ± 0.32	1.08 ± 0.24	1.10 ± 0.44
*Ruminococcaceae* (family)	0.98 ± 0.17		1.41 ± 0.23	2.07 ± 0.38	1.74 ± 0.28	1.43 ± 0.12	2.06 ± 0.30
*Prevotellaceae* (family)	2.36 ± 0.37		0.55 ± 0.19^**^	0.43 ± 0.11	0.74 ± 0.11	0.54 ± 0.11	0.40 ± 0.08
*Clostridiales_vadinBB60_group* (family)	1.20 ± 0.19		1.59 ± 0.27	1.04 ± 0.16	1.63 ± 0.46	1.19 ± 0.25	1.45 ± 0.39
*Bacteroides* (genus)	5.13 ± 0.68		32.29 ± 3.81^**^	30.68 ± 4.33	24.70 ± 2.41	27.94 ± 1.94	20.52 ± 3.28^#^
*Muribaculaceae* (genus)	32.22 ± 2.80		18.75 ± 2.82^**^	12.93 ± 2.46	15.82 ± 3.10	17.12 ± 3.00	20.74 ± 3.60
*Helicobacter* (genus)	1.68 ± 0.57		9.09 ± 1.90^*^	10.18 ± 2.85	14.04 ± 2.67	10.73 ± 1.83	11.99 ± 2.63
*Rikenellaceae_RC9_gut_group* (genus)	20.01 ± 1.89		1.61 ± 0.28^**^	1.14 ± 0.55	1.07 ± 0.17	3.09 ± 1.21	3.55 ± 1.37
*Parabacteroides* (genus)	1.31 ± 0.18		5.98 ± 0.53^**^	4.62 ± 0.95	4.86 ± 0.64	5.50 ± 0.90	5.36 ± 1.03
*Alistipes* (genus)	8.80 ± 0.56		1.29 ± 0.22^**^	0.43 ± 0.16^##^	1.21 ± 0.09	1.28 ± 0.27	1.40 ± 0.09
*Lachnospiraceae_UCG-001* (genus)	0.41 ± 0.22		1.02 ± 0.31	1.65 ± 0.59	3.52 ± 0.70^##^	1.79 ± 0.92	1.60 ± 0.38
*Odoribacter* (genus)	4.29 ± 0.37		0.92 ± 0.27^**^	1.06 ± 0.34	1.42 ± 0.32	1.08 ± 0.24	1.10 ± 0.44
*[Eubacterium]_fissicatena_group* (genus)	0.04 ± 0.02		1.63 ± 0.31^**^	0.69 ± 0.11^#^	1.36 ± 0.49	1.32 ± 0.29	1.18 ± 0.40
*Blautia* (genus)	0.50 ± 0.20		0.74 ± 0.25	1.74 ± 0.55	0.81 ± 0.11	0.70 ± 0.16	0.5 ± 0.11

Compared with the blank group

^*^ *P* < 0.05

^**^ *P* < 0.01 compared with the model group

^#^ *P* < 0.05

^##^ *P* < 0.01

### FRZ-mediated alteration of fecal metabolites of NOD mice

**Fig. 5:**
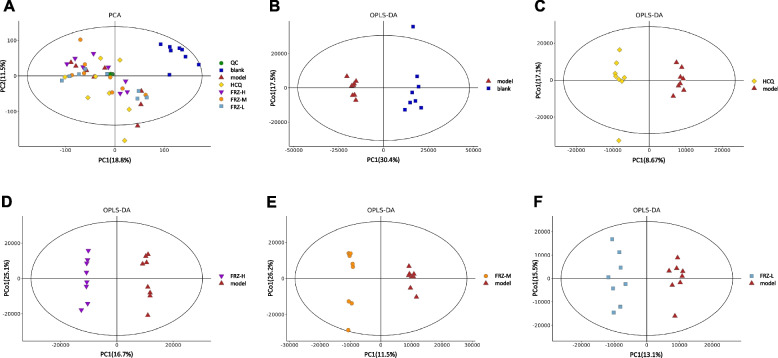
Results from the principal component analysis (PCA) model. **A** Score plots of the PCA model among QC and samples in six groups. **B**–**F** OPLS-DA score graph for the blank and model groups (**B**), model and HCQ groups (**C**), model and FRZ-H groups (**D**), model and FRZ-M groups (**E**), and model and FRZ-L groups (**F**)

XCMS software (SIMCA-P 14.1) was used to extract the metabolite ion peaks of all samples. A total of 4553 and 7179 peaks were obtained in the negative and positive ion mode, respectively. Through the pareto-scaling conversion of all peaks, we obtained a principal component analysis (PCA) model. The QC samples were clustered closely in the positive and negative ion modes, indicating the good reproducibility of the experiments (Fig. 5A). Subsequently, orthogonal partial least squares-discriminant analysis (OPLS-DA) was used to analyze the different metabolites among the six groups. A distinct separation was observed between the blank and model groups, model and HCQ groups, model and FRZ-H groups, model and FRZ-M groups, and model and FRZ-L groups. These differences suggest that the concentration levels of the metabolites in the six groups were significantly different (Fig. 5B-F).

**Fig. 6:**
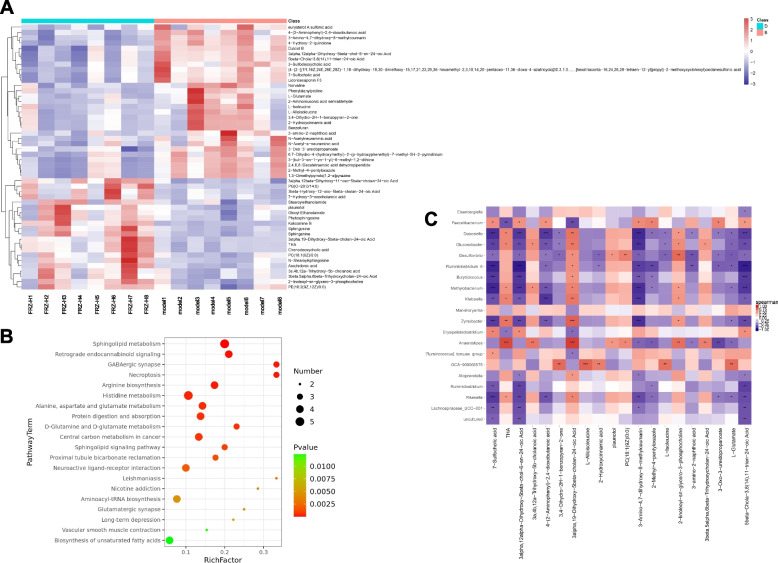
Heat map (**A**), scatterplot (**B**), and Pearson correlation analysis (**C**) of significantly differential metabolites enriched KEGG pathways regulated by FRZ-H for 10 weeks. **A** In the heat map, the x axis indicates the sample name, and the y axis indicates the differential metabolite. Low and high abundance of metabolites is indicated in blue and red, respectively. **B** The numbers of metabolites are indicated by the dot size in the scatterplot. **C** Pearson correlation analysis between fecal metabolites and microbial genera after of high dose of FRZ administration for 10 weeks; ^*^ *P* < 0.05, ^**^ *P* < 0.01 and ^***^ *P* < 0.001

Based on the difference between the blank and model groups, the model and HCQ groups, and the model and FRZ-H groups of the OPLS-DA model, variable values were set at VIP > 1, *P* < 0.05, and fragmentation score > 50 to assess the differential metabolites. The differential metabolites that were identified were 317 (156 downregulated and 161 upregulated) between the blank and model groups, 30 (23 downregulated and 7 upregulated) between the model and HCQ groups, and 109 (47 downregulated and 62 upregulated) between the model and FRZ-H groups (Table S1-3). To show differences in metabolite expression, we performed hierarchical clustering analysis for changes in metabolites between the model and FRZ-H groups (Fig. 6A). In the figure, the abscissa indicates the sample names, and the ordinate indicates the differential metabolites. Colors ranging from blue to red color represent the low to high expression, respectively, of metabolites. Based on the differential metabolites, the KEGG analyses were conducted to discover the most relevant metabolic pathways induced by high dose of FRZ. The top seven significantly enriched pathways (*P* < 0.05) were sphingolipid metabolism, retrograde endocannabinoid signaling, GABAergic synapse, Necroptosis, Arginine biosynthesis, histidine metabolism, and alanine, aspartate, and glutamate metabolism (Fig. 6B).

### Association analysis of microbial diversity with metabolites

Pearson’s correlation analysis was used to determine the correlations between gut microbiota and fecal metabolite profiles induced by high doses of FRZ. The phylum *Proteobacteria* was significantly negatively related to THA and 3alpha,19-dihydroxy-5beta-cholan-24-oic acid. The results of the association analysis between the microbial genera and top 20 metabolites are presented in Fig. 6C. *Lachnospiraceae*_UCG − 001(mainly negative), *Rikenella* (mainly negative), *Ruminiclostridium* (mainly negative), *Alloprevotella* (mainly negative), GCA-900,066,575 (mainly positive), [*Ruminococcus*]_torques_group (mainly positive), *Anaerostipes* (mainly negative), *Erysipelatoclostridium* (mainly positive), *Zymobacter* (mainly negative), *Klebsiella* (mainly negative), *Methylobacterium* (mainly negative), *Butyricicoccus* (mainly negative), *Ruminiclostridium*_6 (mainly negative), *Desulfovibrio* (mainly negative), *Gluconobacter* (mainly negative), *Dubosiella* (mainly negative), and *Faecalibacterium* (mainly positive) were closely associated with numerous differential fecal metabolites. The changes in *Eisenbergiella* were only significantly positively related to changes in 5beta-chola-3,8(14),11-trien-24-oic acid (*P* < 0.05), and [*Ruminococcus*]_torques_group was significantly related to 7-sulfocholic acid (*P* < 0.05) and 3alpha,19-dihydroxy-5beta-cholan-24-oic acid (*P* < 0.05). The results at the family and species levels are shown in Supplementary Figure S1.

## Discussion

SS is a chronic inflammatory autoimmune disease that is divided into primary and secondary types, mainly involves exocrine glands. Clinical symptoms of SS often include dry eyes, conjunctivitis, and dry mouth. Internal organ involvement can include liver, lungs, kidneys, pancreas, nervous system, and blood system [1]. Recent studies have shown that the pathogenesis of SS is a combination of genetic, viral, or bacterial infections and abnormal hormone levels, which lead to immune disorders. Currently, there are no radical treatments for SS. Treatment is typically designed to improve clinical symptoms and control or prolong the progression of tissue and organ damage caused by the immune response and secondary infection [19]. When multiple tissues or organs are involved in SS, immunosuppressants or hormones are added to delay autoimmune response. However, the long-term use of immunosuppressants or hormones causes many adverse reactions [20]. This decrease the quality of life of patients with SS. In recent years, our previous clinical studies have confirmed that FRZ was effective in improving the clinical symptoms and signs, including dry eyes, dry mouth, dry skin, fatigue, and joint pain in SS patients [7–9]. Besides, in vitro cell experiments and animal experiments also showed significant roles of FRZ in the treatment of SS [11–13, 21]. Study the mechanisms of FRZ may facilitate the development of new pharmaceutical strategies for the treatment of SS.

Besides, the relationship between gut microbiota and diseases is attracting more and more attention. Recent research has shown that intestinal microbiota can affect the balance between proinflammatory and anti-inflammatory immune responses, leading to prolong the progression of rheumatic diseases [22]. A close connection between the intestinal microbiota and immune cells of the intestinal mucosa has been demonstrated. Due to the abnormal function of exocrine glands, the secretion of intestinal mucus is significantly reduced, and the protection and renewal function of the gastrointestinal epithelium are affected, leading to a disorder of the microbiota-host immune system in SS patients [23]. In addition, microbiota can regulate the permeability of the gut and certain species can promote “gut leakage,” in which microbial-related metabolites leave the gut and enter the bloodstream. In response, the body produces cytokines and other mediators that efficiently initiate inflammatory response [24]. Similarly, cells within intestinal epithelial tissues deliver bacterial metabolites to immune cells, promoting inflammation on systemic and local scales. Persistence of the condition may lead to chronic or subacute inflammation, which may lead to the development of the disease [25]. Previous studies have shown differences in the gut microbiome taxa in SS patients compared with healthy controls, with significantly decreased relative abundance of *Parabacteriodes* and *Faecalibacterium*, and increased abundance of *Streptococcus*, *Blautia*, *Escherichia*, and *Pseudobutyrivibrio* [26]. Mandl et al. (2017) also reported a decreased relative abundance of *Bifidobacterium*, *Alistipes*, and *Faecalibacterium* in SS [27].

Studies on the gut microbiota in SS patients have mostly been based on small sample numbers. The gut microbiota changes in patients with SS mainly show this decrease in microbiota diversity, and there is no consistent conclusion regarding specific microbiota lineage alterations. In one study, the gut microbiota in SS patients was significantly different from that in healthy controls and specifically showed a decrease in bacteria producing protective butyrate and an increase in proinflammatory harmful genera [28]. In the present study, NOD mice were continuously treated with high, medium, and low doses of FRZ for 10 weeks. The volume of drinking water of mice in the FRZ treatment and HCQ groups decreased, while the submandibular gland index was increased compared to the model group. The histology of the submandibular glands was checked using H&E staining; NOD mice displayed pathologies that included disappearance of the lobular structure of the submandibular glands and infiltrating lymphocytes. In addition, the pathological phenomena of the submandibular gland tissue in the HCQ-treated group and FRZ-H and FRZ-M groups were alleviated. At the same time, ELISA results showed that the high dose of FRZ significantly decreased the levels of IL-6, TNF-α, and IL-17 A while increasing the levels of IL-10, suggesting the effect of FRZ in regulating the inflammatory and anti-inflammatory cytokines in NOD mice. Dysregulation of the gut microbiota can regulate systemic inflammation, which also reduces beneficial gut bacteria and promotes the growth of commensal bacteria with the potential to become pathogenic [29]. Chronic autoimmune inflammation has been demonstrated in SS patients and in animal models of SS, and many cytokines play an important role in regulating the inflammatory response [30]. Cytokines secreted from helper T cells, such as Th1, Th2, and Th17, as well as B cells, are involved in the inflammatory response of SS. Among them, TNF-α can promote the activation of both Th1 and Th2 cells, IL-17 can promote the early inflammatory response induced by T cells, and IL-6 can promote the proliferation and differentiation of B cells [30]. IL-10, a key anti-inflammatory mediator secreted primarily by monocytes, has multiple effects on immune regulation and inflammation, and can protect the host from overresponse to pathogens and microbiota [31]. In patients with primary SS, the integrity of the gastrointestinal epithelium and its barrier function may be altered by inflammation and reduced exocrine glandular secretions, thereby changing the interactions of microbiota-host immune system, with the dysfunction of exocrine gland driven by high levels of IL-6, IL-17 and TNF-α [32]. The gut microbiota is involved in maintaining the immune response balance between Th17 cells and Treg cells on the mucosal surface and serves as a trigger for inducing autoimmunity. A study exploring the role of butyrate (a gut microbiota metabolite) in regulating B cells in NOD mice demonstrated that butyrate may ameliorate SS via reciprocal regulation of IL-10 and IL-17-producing B cells [33]. It is worth noting that segmented filamentous bacterial antigens presented by intestinal dendritic cells are able to drive the differentiation of mucosal Th17 [34]. IL-17 regulates the expression of tight junction proteins, which are important for maintaining mucosal permeability in epithelial cells [35]. Collected studies confirmed the important role of cytokines in regulating the inflammatory response in SS, and FRZ could regulate these cytokines to reduce the inflammatory responses.

In addition, our findings demonstrated the altered composition of the gut microbiota and its metabolites in NOD mice after FRZ treatment. This is the first study to investigate the correlation between gut microbiota and fecal metabolites in NOD mice. We found that microbial richness in NOD mice changed significantly after 10 weeks of FRZ-H and FRZ-L treatment. Moreover, the composition of the gut microbial community was significantly different in NOD mice after FRZ therapy compared with those without FRZ therapy. Analysis of the structure of the gut microbiota at the phylum level revealed that Firmicutes (25.12%) and Bacteroidetes (64.10%) were the most abundant in the model group. The ratio of Firmicutes to Bacteroidetes (F/B ratio) is commonly used to assess the imbalance of gut microbiota. The F/B ratio is decreased in many diseases, such as inflammatory bowel disease (IBD), rheumatoid arthritis (RA), and systemic lupus erythematosus (SLE) [36, 37]. In the present study, the F/B ratio in the FRZ treatment groups (0.58, 0.46, and 0.54 in the FRZ-H, FRZ-M, and FRZ-L group, respectively) was higher than that in the model group (0.39). These findings suggest that FRZ could improve the balance of gut microbiota in NOD mice by increasing the abundance of *Firmicutes*, thereby inhibiting the pathogenic effects of Bacteroides.

At the genus level, the increased abundance of *Lachnospiraceae_UCG-001* induced by FRZ was statistically significant. Similar results were reported in an intestinal microecology study in primary SS patients treated with the Yangyin Yiqi Huoxue decoction (YYHD). The authors also observed a significant increase in the proportion of *Lachnospiraceae* after YYHD treatment. These findings suggest that *Lachnospiraceae_UCG-001* may be a potentially helpful bacterium in the metabolism of various carbohydrates and the fermentation of glucose products, including formic acid, lactic acid and acetic acid [38, 39]. The FRZ-induced upregulation of *Lachnospiraceae_UCG-001* may be beneficial to host health.

Although several studies have reported gut dysbiosis in SS, the role of bacteria or metabolites in SS remains unknown. Gut microbiota influences host immunity by producing microbial metabolites. In this study, the increased abundance of *Proteobacteria* treated with FRZ was significantly positively associated with THA and 3alpha,19-dihydroxy-5beta-cholan-24-oic acid. *Proteobacteria* contain a variety of opportunistic pathogens and are often involved in many diseases [40]. THA is an unsaturated fatty acid and no studies have shown its association with immune or enteric diseases. 3Alpha,19-dihydroxy-5beta-cholan-24-oic acid is associated with the metabolic pathway of conjugated bile acids. The bile acid-gut microbiota axis plays an important role in patients with IBD) patients [41]. Enteric infections with bacteria that include *Bacillus comma* and *Clostridioides difficile*, and parasite *Entamoeba* as the enteropathogen are regulated by the metabolism of bile acids [42]. At the genus level, the increased abundance of *Lachnospiraceae*_UCG − 001 induced by FRZ was negatively related to 7-sulfocholic acid, 3alpha,12alpha-dihydroxy-5beta-chol-6-en-24-oic acid, 3-amino-4,7-dihydroxy-8-methylcoumarin, and 5beta-chola-3,8(14),11-trien-24-oic acid. The decreased abundance of *Erysipelatoclostridium* induced by FRZ was positively related to 7-sulfocholic acid, 3alpha,12alpha-dihydroxy-5beta-chol-6-en-24-oic acid, and 5beta-chola-3,8(14),11-trien-24-oic acid, and negatively related to THA and 3alpha,19-dihydroxy-5beta-cholan-24-oic acid. Except for 3-amino-4,7-dihydroxy-8-methylcoumarin, the other four metabolites were classified as bile acids and their derivatives. 3-Amino-4,7-dihydroxy-8-methylcoumarin is a hydroxycoumarin, specifically 4,7-dihydroxycoumarin with an additional amino and methyl substituent at position 3 and 8, respectively.

According to the changes in the microbial community, the fecal metabolites also changed in NOD mice after 10 weeks of FRZ treatment. Differential fecal metabolites were enriched in the pathways of sphingolipid metabolism, retrograde endocannabinoid signaling, GABAergic synapse, necroptosis, arginine biosynthesis, histidine metabolism, and alanine, aspartate, and glutamate metabolism. Sphingolipid metabolites are essential signaling molecules that regulate cell growth, survival, immune cell trafficking, and vascular and epithelial cell integrity. These metabolites are particularly serious in inflammation, autoimmunity, and cancer [43, 44]. Finlimod is a drug that targets sphingosine 1-phosphate. Finlimod is used to treat multiple sclerosis [45]. Retrograde endocannabinoid signaling can regulate many physiological and behavioral functions. Zhang et al. (2014) reported the association of the retrograde endocannabinoid signaling pathway with susceptibility to RA [46]. GABAergic synapses play a key role in the physiological and cellular immune regulation of the central nervous system, especially when maintaining the balance between neural network excitation and suppression. Breaking this balance is related to neuropsychiatric and autoimmune disorders [47]. Necroptosis is programmed cell death regulated by receptor-interacting protein kinase-1 (RIPK1), RIPK3, and mixed lineage kinase-like [48]. The morphological features of necroptosis have been extensively investigated, including inflammation in RA through the release of cellular contents [49]. Arginine, as a component of salivary mucin, can accompany salivary mucin adhesion to the tooth surface and, under the action of the arginine deiminase system, decompose to produce ammonia, carbon dioxide, and ATP, regulating the balance of oral microflora [50]. Histidine is an essential amino acid that can reduce oxidative stress, inflammation, and metabolic syndrome [51]. Acrolein-activated 92 kDa metalloproteinase-9 is elevated by approximately 2.4-fold in the saliva of primary SS patients. This metalloproteinase is involved in tissue damage in patients with primary SS and is regulated by both cysteine and histidine [52]. A study of untargeted serum metabolomics and potential biomarkers in patients with SS showed that the alanine, aspartate, and glutamate metabolism pathways were significantly altered [53]. These findings are consistent with the results of the present study and indicate that the enriched metabolic pathways in NOD mice might be dysregulated after exposure to FRZ.

The limitation of this study is that we did not detect the effects of the active components in FRZ and determine whether they have the same or similar effects on the microbial and metabolic profiles of NOD mice. Differential gut microflora, metabolites, and specific signal pathway responses to FRZ will be further investigated in our laboratory.

## Conclusion

From the above experiments, the effects of FRZ on the gut microbiota and fecal metabolites were explored in NOD mice. We concluded that FRZ treatment could reduce the inflammatory responses in NOD mice by regulating the gut microbiota, fecal metabolites, and their correlation to emerge a therapeutic effect on mice with SS. Through 16 S rRNA gene sequencing analysis, we found that FRZ downregulated *Bacteroidaceae* (family) and *Bacteroides* (genus), and upregulated *Lachnospiraceae_UCG-001* (genus). Using fecal metabolomics, the fecal metabolites were significantly changed after FRZ treatment, and 47 downregulated and 62 upregulated metabolites were identified in the FRZ-H group. Combined gut microbiota and fecal metabolites analysis, we further discovered that the regulated genera were closely associated with many differential fecal metabolites. These results might provide scientific evidence for FRZ treatment of SS and the use of gut microbiotas as drug targets to treat SS.

## Supplementary Information

**Additional file 1: Table S1. **Significantly changed metabolites found in LC/MS-based metabolomic profiling (blank group vs model group). **Table S2.** Significantly changed metabolites found in LC/MS-based metabolomic profiling (model group vs HCQ group). **Table S3.** Significantly changed metabolites found in LC/MS-based metabolomic profiling (model group vs FRZ-H group).

**Additional file 2: Supplementary Figure 1. **Pearson correlation analysis between fecal metabolites and microbial family (A) and species (B) after of high dose of FRZ administration for 10 weeks; ^*^P<0.05,^**^P<0.01 and ^***^P<0.001.

## Data Availability

The raw 16 S rRNA sequencing dataset for this study has been deposited in the Sequence Read Archive (SRA) under accession number PRJNA927120 (https://www.ncbi.nlm.nih.gov/sra/PRJNA927120). The authors declare that all other data supporting the findings of the study are available in the main text and supplementary materials, or from the corresponding author upon request.
