# Supplementary material for: Gut microbiota combined with fecal metabolomics reveals the effects of FuFang Runzaoling on the microbial and metabolic profiles in NOD mouse model of Sjögren’s syndrome
Source: BMC Complement Med Ther. 2023 Jun 13;23:195. doi: 10.1186/s12906-023-04017-5 (PMC10262465; doi:10.1186/s12906-023-04017-5)
Supplement: Supplementary file 2 — Additional file 2: Supplementary Figure 1. Pearson correlation analysis between fecal metabolites and microbial family (A) and species (B) after of high dose of FRZ administration for 10 weeks; *P<0.05,**P<0.01 and ***P<0.001. [file 12906_2023_4017_MOESM2_ESM.docx]

**
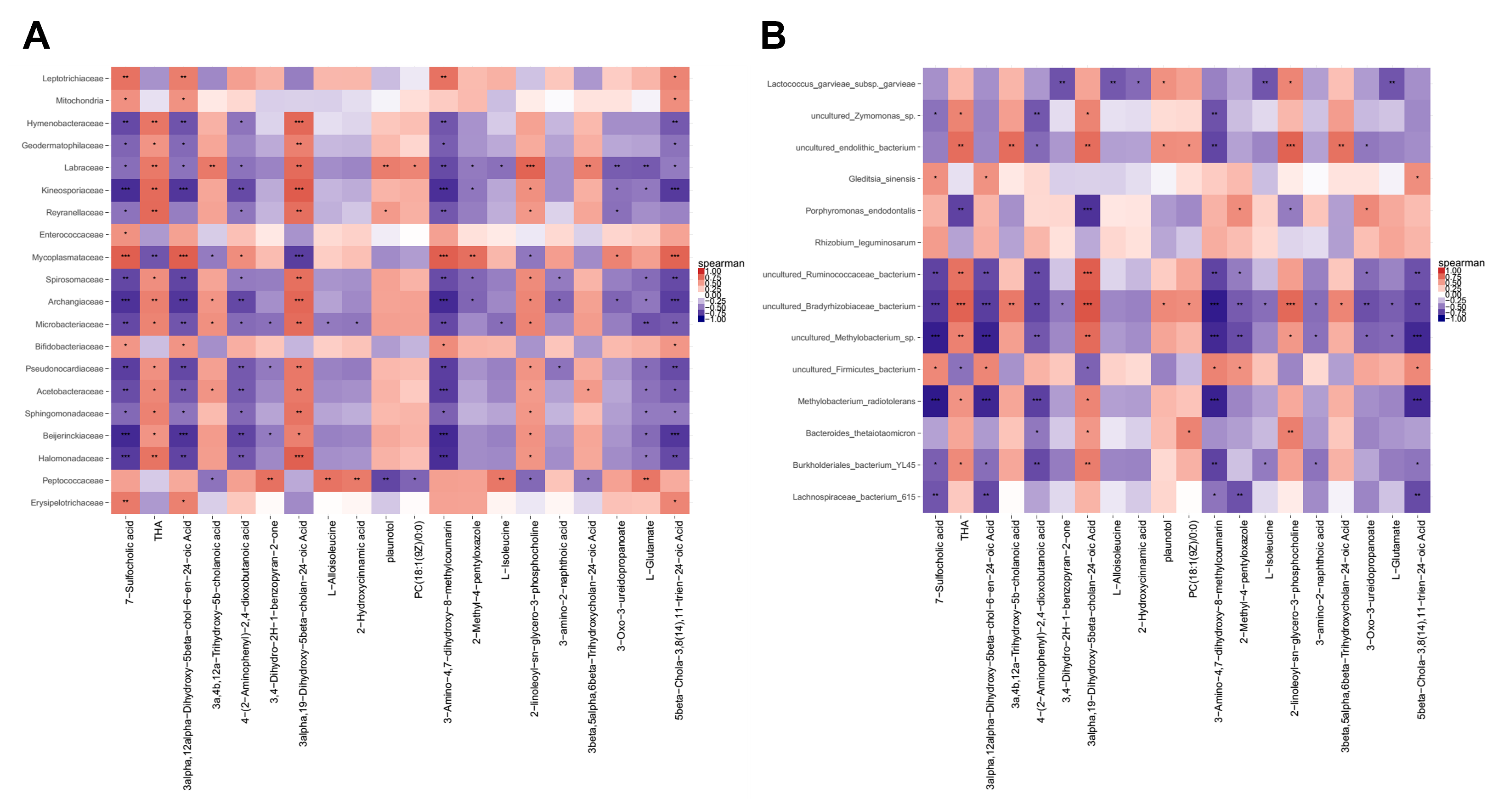
**

Supplementary Figure 1. Pearson correlation analysis between fecal metabolites and microbial family (A) and species (B) after of high dose of FRZ administration for 10 weeks; ^*^*P*<0.05, ^**^*P*<0.01 and ^***^*P*<0.001.
